# Supplementary material for: Injectable hydrogel electrodes as conduction highways to restore native pacing
Source: Nat Commun. 2024 Jan 2;15:64. doi: 10.1038/s41467-023-44419-0 (PMC10762156; doi:10.1038/s41467-023-44419-0)
Supplement: Supplementary file 1 — Supplementary Information [file 41467_2023_44419_MOESM1_ESM.pdf]

Supplementary Information

Injectable hydrogel electrodes as conduction highways to restore native pacing

Authors: Gabriel J. Rodriguez-Rivera,[1] Allison Post,[2] Mathews John,[2] Skylar Buchan,[2] Drew Bernard, [2] Mehdi Razavi,[2,3*] Elizabeth Cosgriff-Hernandez[4*]

[1]McKetta Department of Chemical Engineering, The University of Texas at Austin; Austin, TX 787212, USA.
[2]Electrophysiology Clinical Research and Innovations, Texas Heart Institute; Houston, TX 77030, USA.
[3]Division of Cardiology, Department of Medicine, Baylor College of Medicine; Houston, TX 77030, USA.
[4]Department of Biomedical Engineering, The University of Texas at Austin; Austin, TX 78712, USA.

*Corresponding authors:
Elizabeth Cosgriff-Hernandez: cosgriff.hernandez@utexas.edu
Mehdi Razavi: mehdirazavi1@gmail.com

This document includes the following Supplementary Information:

Supplementary Figures 1 – 33

Supplementary Tables 1 – 3

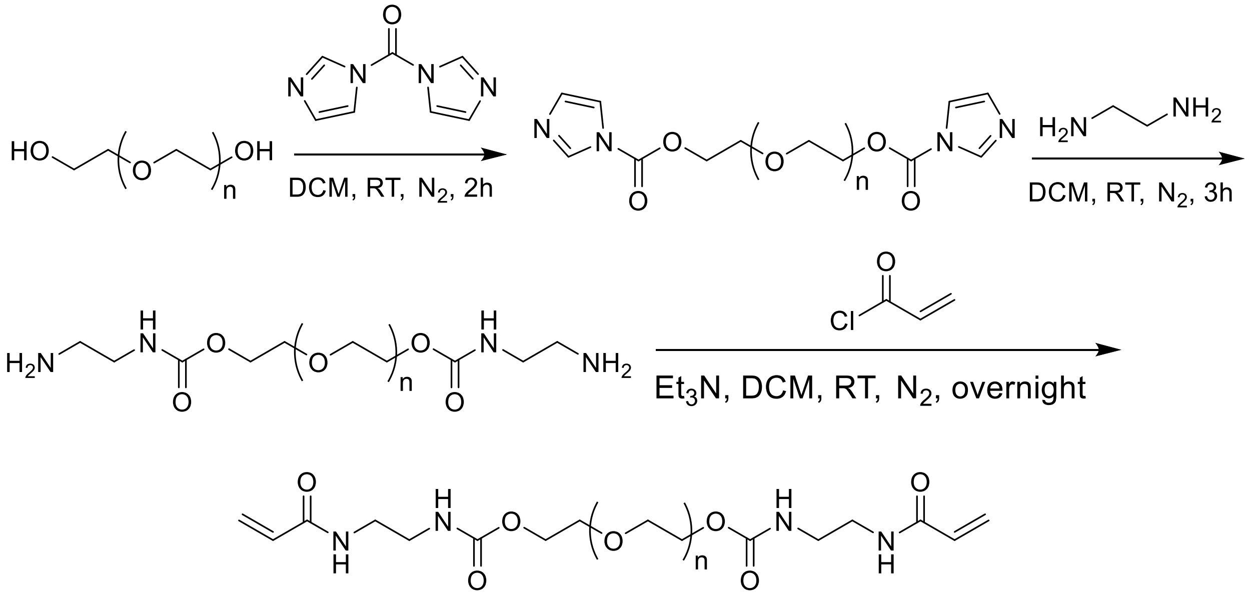

Supplementary Figure 1. Synthetic route for PEUDAm.

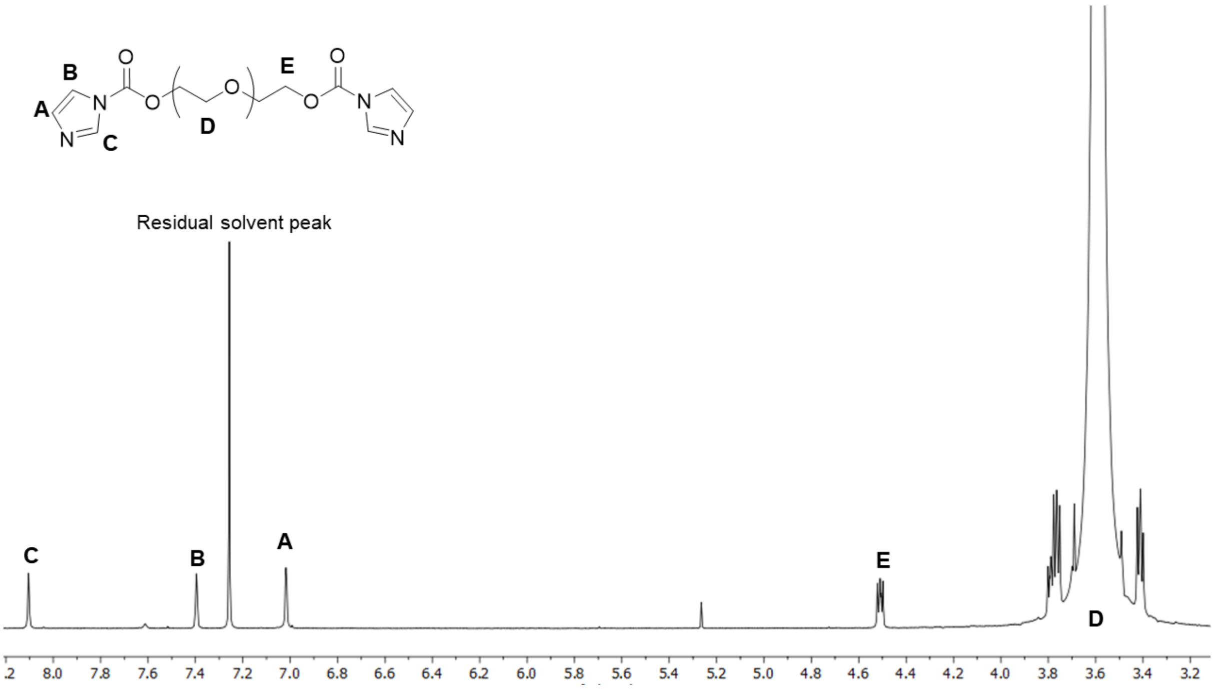

Supplementary Figure 2. H^1 NMR Spectra of PEG-CDI. Source data are provided as a Source Data file.

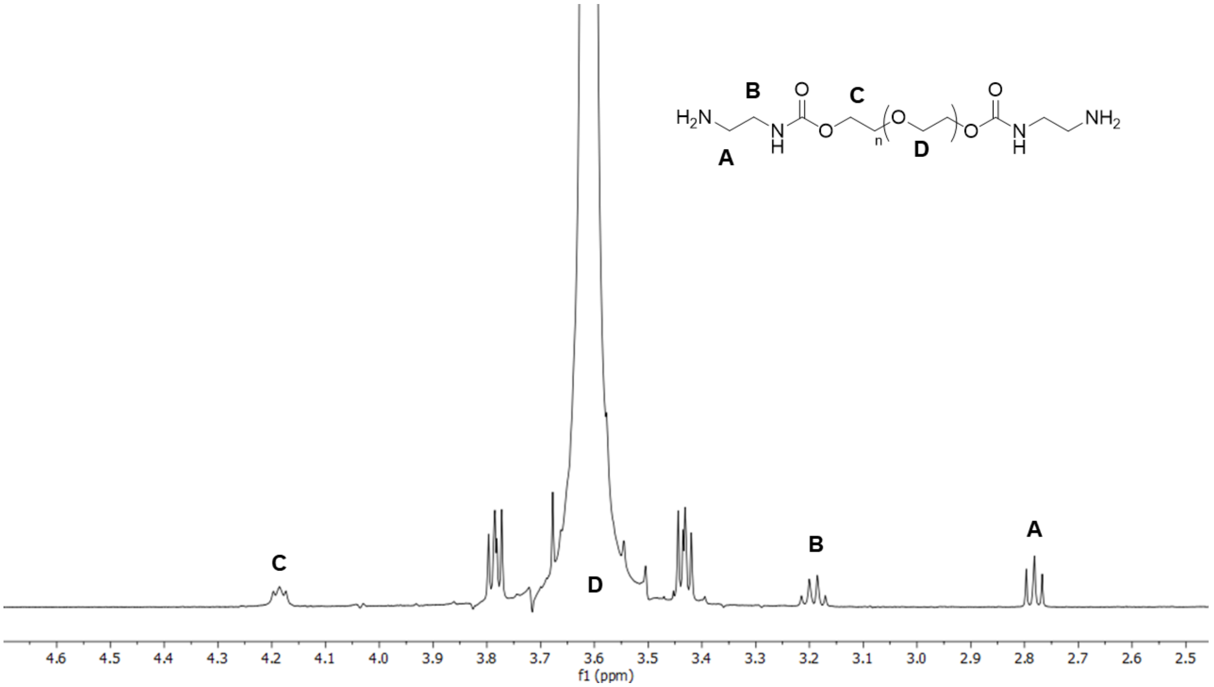

Supplementary Figure 3. H^1 NMR Spectra of PEG-EDA. Source data are provided as a Source Data file.

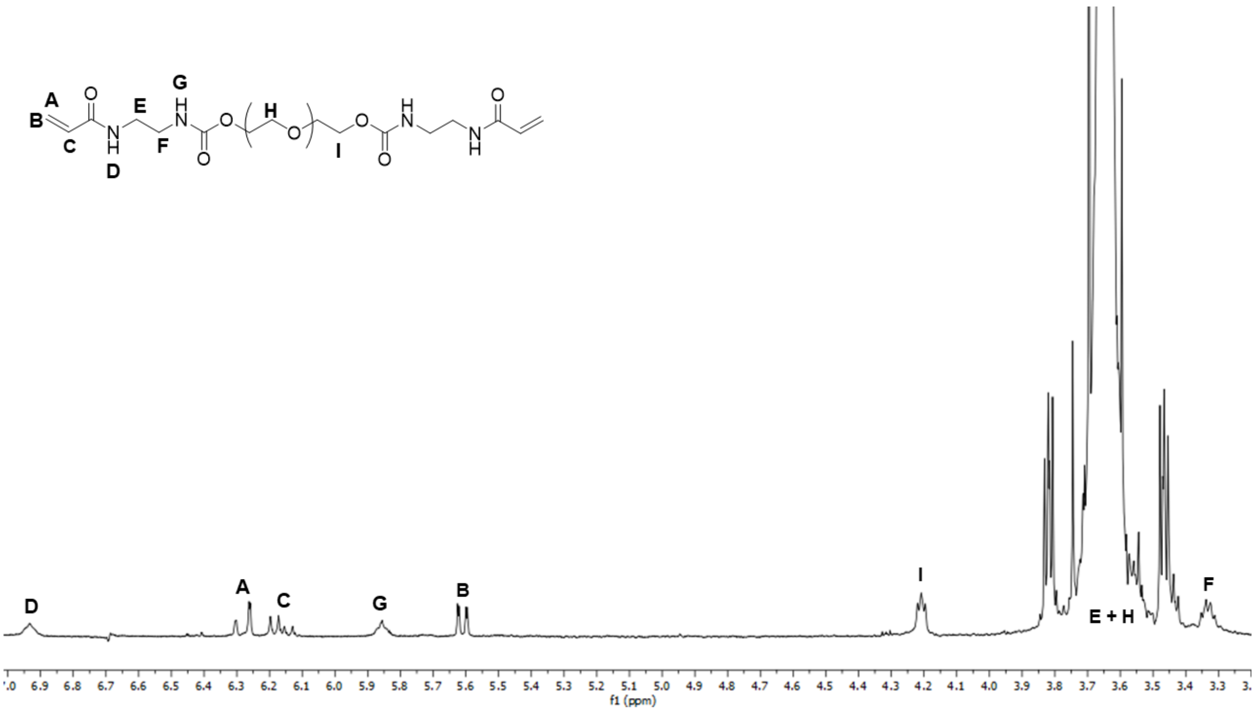

Supplementary Figure 4. H^1 NMR Spectra of PEUDAm. Source data are provided as a Source Data file.

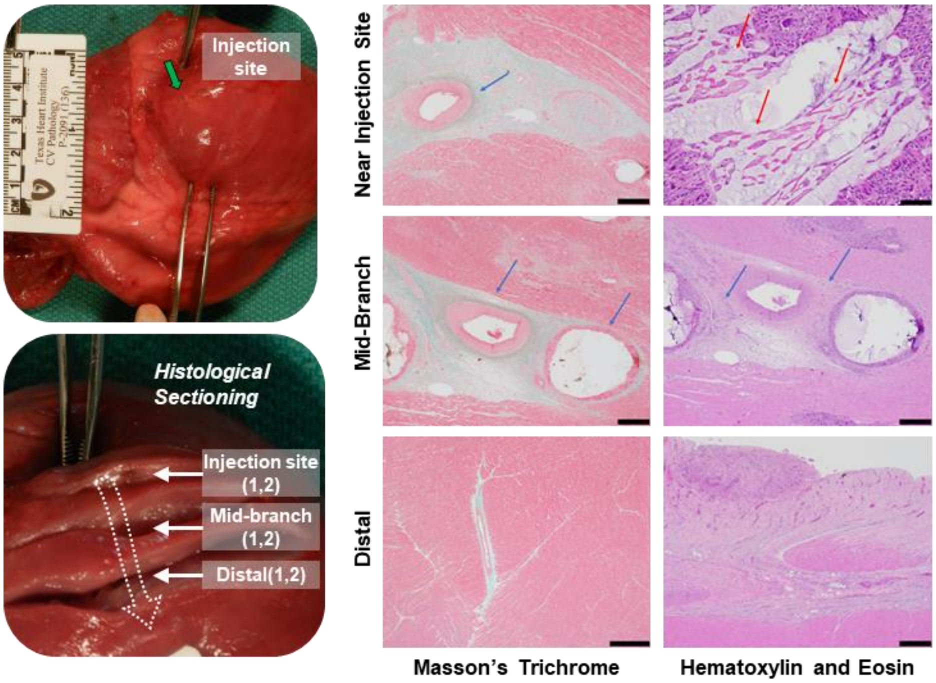

Supplementary Figure 5. *In vivo* assessment of injectable electrode in coronary vein (middle cardiac vein) of a porcine model. Host response after 2 weeks implantation in MCV using slices of the location proximal to the injection site, middle of the vein, and distal to the injection site (green arrow). The white arrow indicates the approximate location of the hydrogel in the vein. The response includes damage induced at the hydrogel injection site, with 1) moderate perivascular and interstitial fibrosis with focal replacement fibrosis (blue arrows), and 2) fibrosing epicarditis with foreign body giant cell reaction (red arrows). Mid branch of the hydrogel injection show 3) mild perivascular and interstitial fibrosis, 4) mild focal replacement fibrosis, and fibrosing epicarditis. Distal branch indicated 5) preserved myocardium with 6) only fibrosing epicarditis with focal extension into myocardium. Scale bars for images 1 through 6 correspond to 400 µm, 75 µm, 400 µm, 400 µm, 1 mm, and 400 µm respectively.

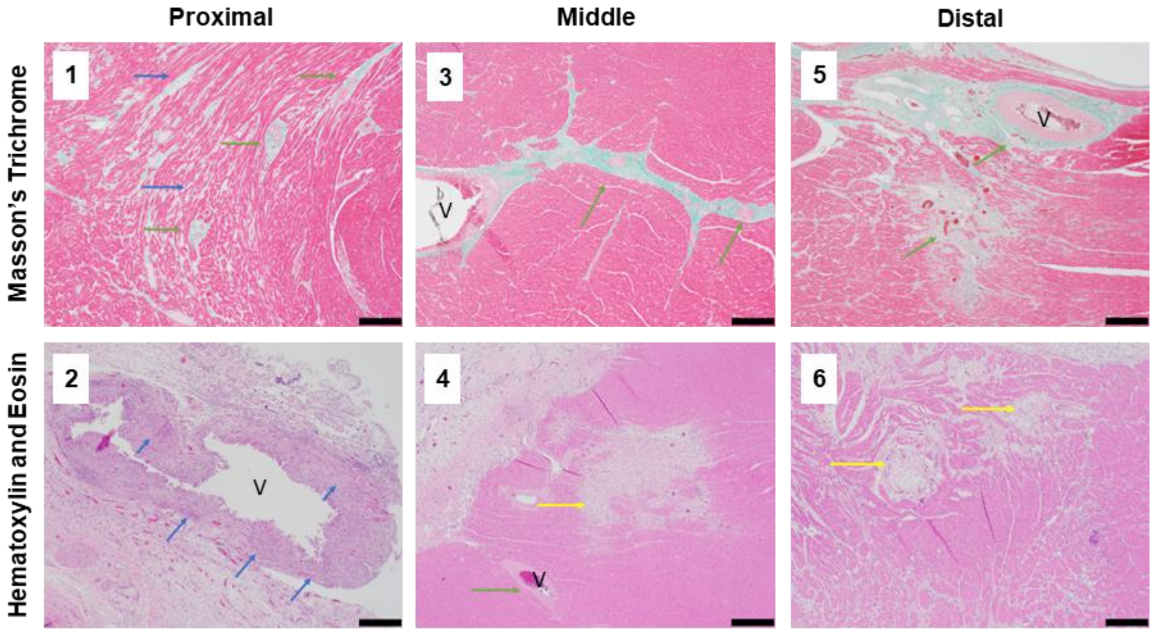

Supplementary Figure 6. *In vivo* assessment of injectable electrode in coronary vein (anterior interventricular vein) of a porcine model (Animal 1). Host response after 4 weeks implantation in AIV using slices of the location proximal to the injection site, middle of the vein (indicated by V), and distal to the injection site. The response includes damage induced at the hydrogel injection site, with 1) mild perivascular (green arrows) and interstitial fibrosis (blue arrows), and 2) extensive vein dilation with transmural chronic inflammation and foreign body giant cell reaction (blue arrows). Mid branch of the hydrogel injection show 3) mild perivascular and interstitial fibrosis (green arrows), 4) and mild focal replacement fibrosis (yellow arrows). The images at the distal branch indicated 5) perivascular and interstitial fibrosis (green arrows) with 6) mild focal extension into myocardium (yellow arrows). Scale bars reflect 400 µm.

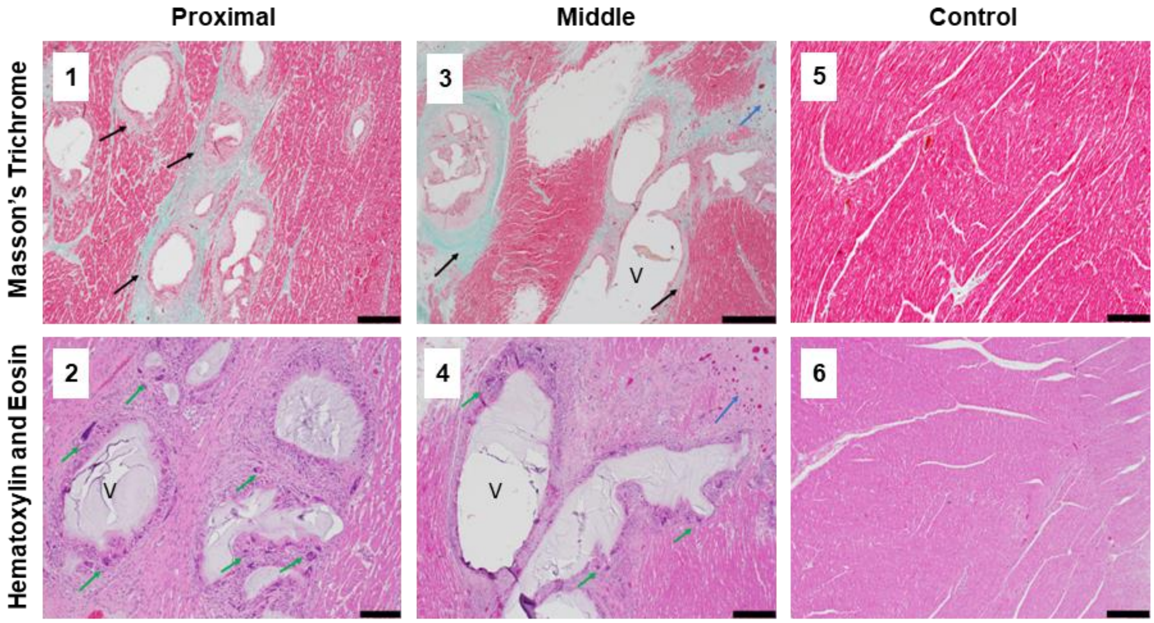

Supplementary Figure 7. ***In vivo*** **assessment of injectable electrode in coronary vein (anterior interventricular vein) of a porcine model (Animal 2).** Host response after 4 weeks implantation in AIV using slices of the location proximal to the injection site, middle of the vein (indicated by V), and the control from an alternate section of the anterior wall. The response includes damage induced at the hydrogel injection site, with 1) moderate perivascular and interstitial fibrosis with focal replacement fibrosis (blue arrows), and 2) fibrosing epicarditis with foreign body giant cell reaction (green arrows). Mid branch of the hydrogel injection show 3) moderate perivascular and interstitial (black arrows) fibrosis, and replacement fibrosis (blue arrows), 4) focal replacement fibrosis, and fibrosing epicarditis. The control images at an alternate section of the anterior wall indicated 5) 6) preserved myocardium. Scale bars for images 1 through 6 correspond to 400 µm, 150 µm, 1 mm, 400 µm, 400 µm, and 400 µm respectively.

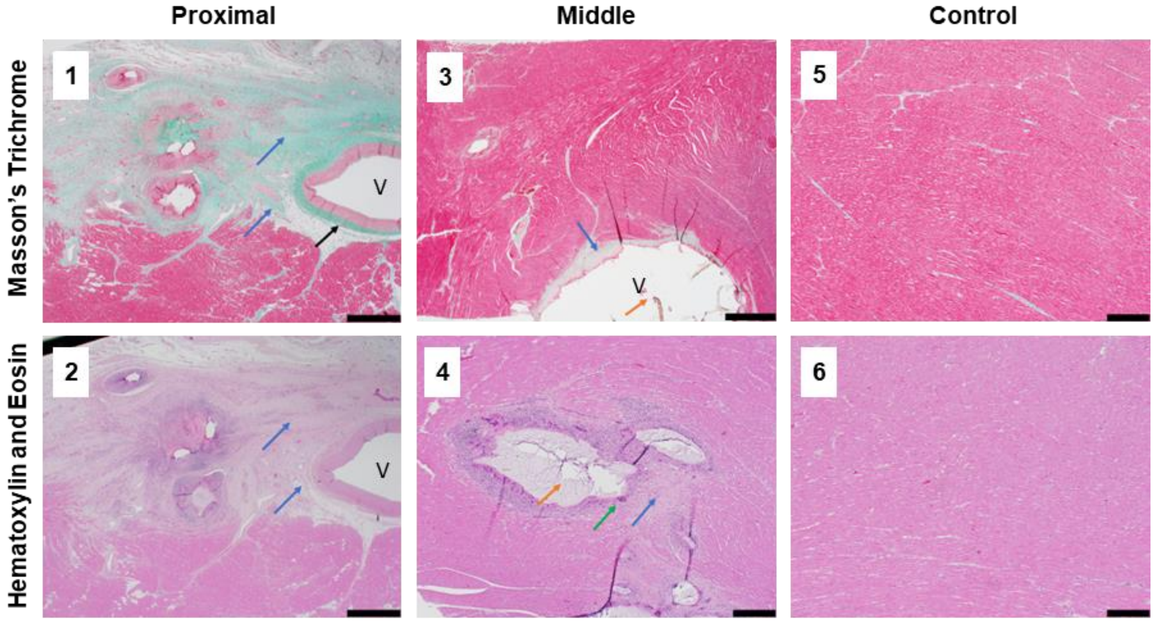

Supplementary Figure 8. *In vivo* assessment of injectable electrode in coronary vein (anterior interventricular vein) of a porcine model (Animal 3). Host response after 4 weeks implantation in AIV using slices of the location proximal to the injection site, middle of the vein (indicated by V), and the control from an alternate section of the anterior wall. The response includes damage induced at the hydrogel injection site, with 1) 2) moderate epicardial (blue arrows) and perivascular fibrosis (black arrows), with focal replacement fibrosis and foreign body giant cell reaction. Mid branch of the hydrogel injection show 3) mild perivascular and interstitial (blue arrows) fibrosis with dilated intramyocardial vessels (orange arrows), and 4) foreign body giant cell reaction (green arrows). The control images at an alternate section of the anterior wall indicated 5) 6) preserved myocardium. Scale bars for images 1 through 6 correspond to 1mm, 1mm, 400 μm, 1.5 mm, 400 μm, and 400 μm respectively.

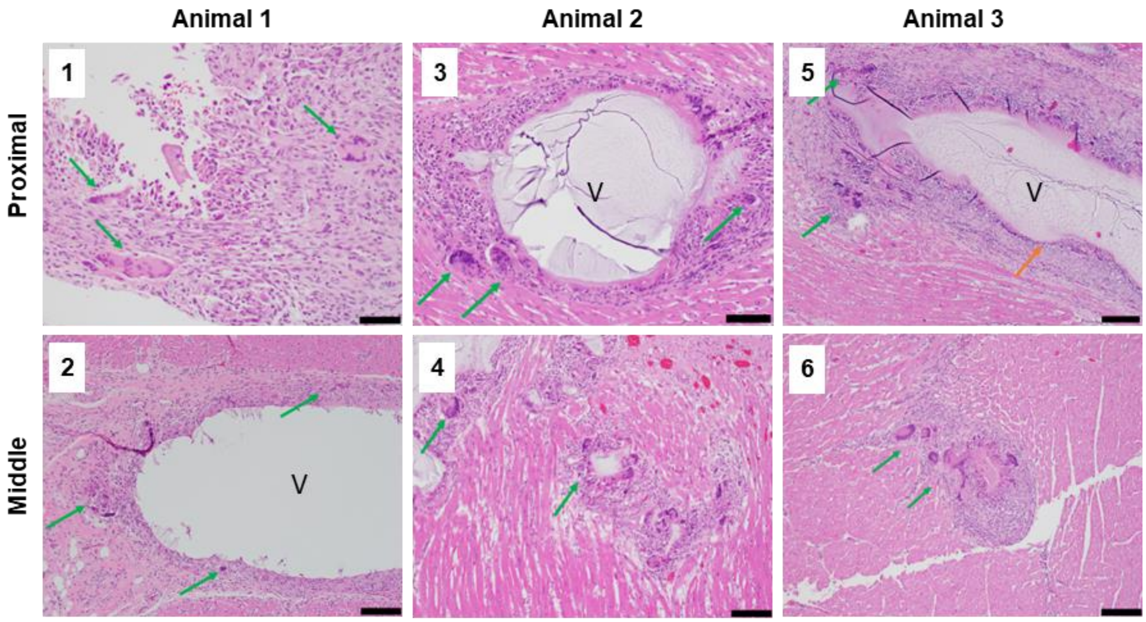

Supplementary Figure 9. High-magnification views of foreign body giant cell reactions to injectable electrodes in coronary vein (anterior interventricular vein). Animal 1 host response after 4 weeks implantation in AIV using slices of the location proximal to the injection site and at the middle of the vein (indicated by V). The response includes damage induced at the hydrogel injection site, with 1) transmural chronic inflammation and foreign body giant cell reaction (green arrows), and 2) mid branch transmural chronic inflammation with foreign body giant cell reaction. Animal 2 host response after 4 weeks implantation proximal to the injection site show 3) epicardial fibrosis with foreign body giant cell reaction, and 4) dilated intramyocardial vessels with foreign body giant cell reaction. Animal 3 host response after 4 weeks implantation proximal to the injection site show 5) 6) dilated intramyocardial vessels (orange arrow) with foreign body giant cell reaction, and. Scale bars for images 1 through 4 correspond to 75 µm, 150 µm, 100 µm, 150 µm, 200 µm, and 150 µm respectively.

A. Time matched ECG tracings. **B.** QRS and vector morphology matched ECG tracings.

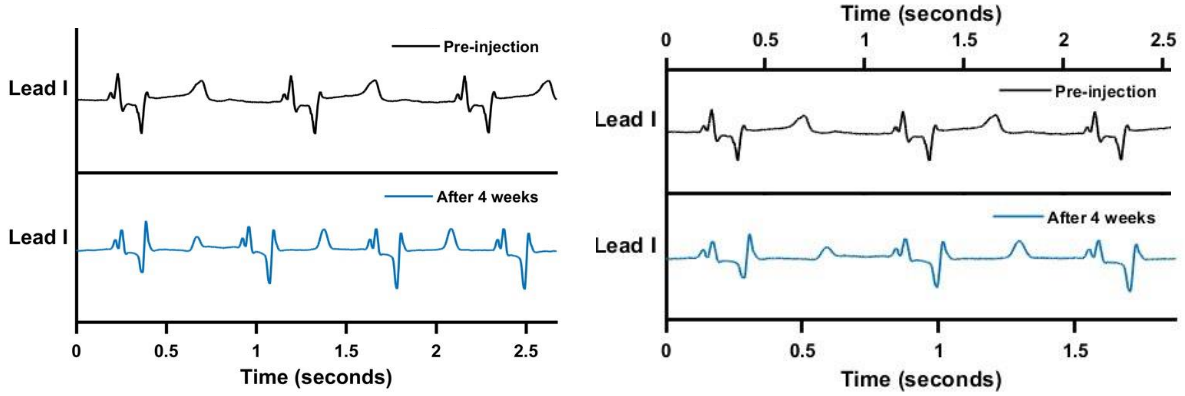

Supplementary Figure 10. *In vivo* assessment of injectable electrode in coronary vein (anterior intraventricular vein) of a porcine model. Animal AIV 1. ECG tracings from lead 1 of the cardiac activity before injection of the hydrogel (black tracing) and 4 weeks after injection (blue tracing). The overall QRS morphology is preserved. Heart rate differs between the two recordings, but this is not indicative of a disease state. Source data are provided as a Source Data file.

A. Time matched ECG tracings with same heart rate.

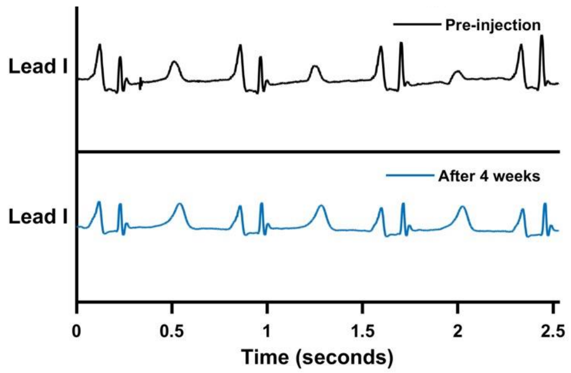

Supplementary Figure 11. ***In vivo*** **assessment of injectable electrode in coronary vein (anterior intraventricular vein) of a porcine model.** Animal AIV 2. ECG tracings from lead 1 of the cardiac activity before injection of the hydrogel (black tracing) and 4 weeks after injection (blue tracing). The overall QRS morphology is preserved. Source data are provided as a Source Data file.

A. Time matched ECG tracings. **B.** QRS and vector morphology matched ECG tracings.

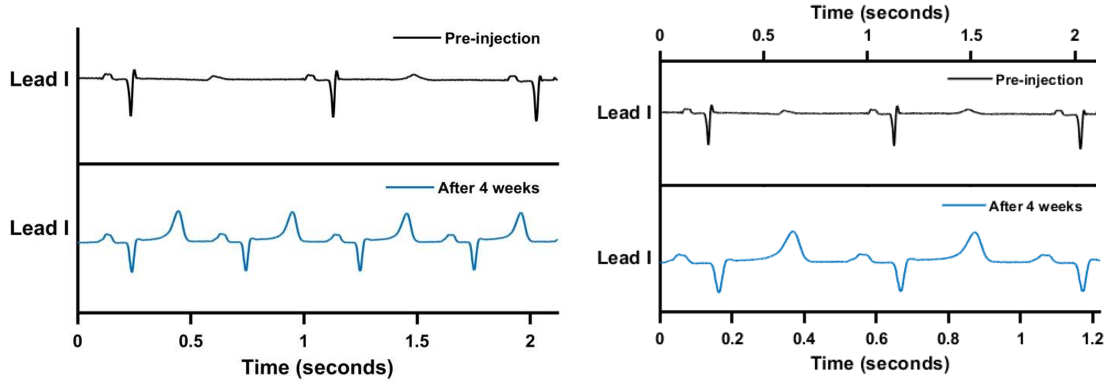

Supplementary Figure 12. *In vivo* assessment of injectable electrode in coronary vein (anterior intraventricular vein) of a porcine model. Animal AIV 3. ECG tracings from lead 1 of the cardiac activity before injection of the hydrogel (black tracing) and 4 weeks after injection (blue tracing). The overall QRS morphology is similar, with nearly identical vectors. Heart rate differs between the two recordings, but this is not indicative of a disease state. Source data are provided as a Source Data file.

A. Time matched ECG tracings. **B.** QRS and vector morphology matched ECG tracings.

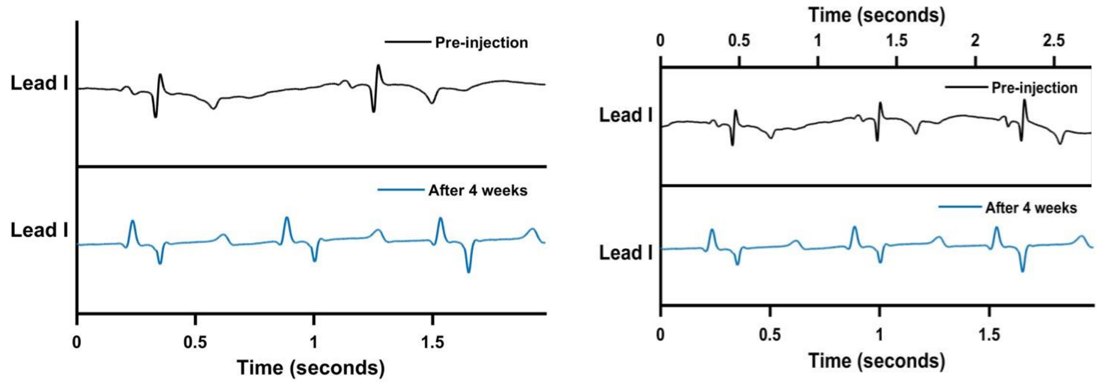

Supplementary Figure 13. *In vivo* assessment of injectable electrode in coronary vein (middle cardiac vein) of a porcine model. Animal MCV 1. ECG tracings from lead 1 of the cardiac activity before injection of the hydrogel (black tracing) and 2 weeks after injection (blue tracing). The overall QRS morphology is similar, with nearly identical vectors. Heart rate differs between the two recordings, but this is not indicative of a disease state. The pre-injection ECG tracing contains low frequency baseline drift which is not reflective of the model’s electrophysiology. Source data are provided as a Source Data file.

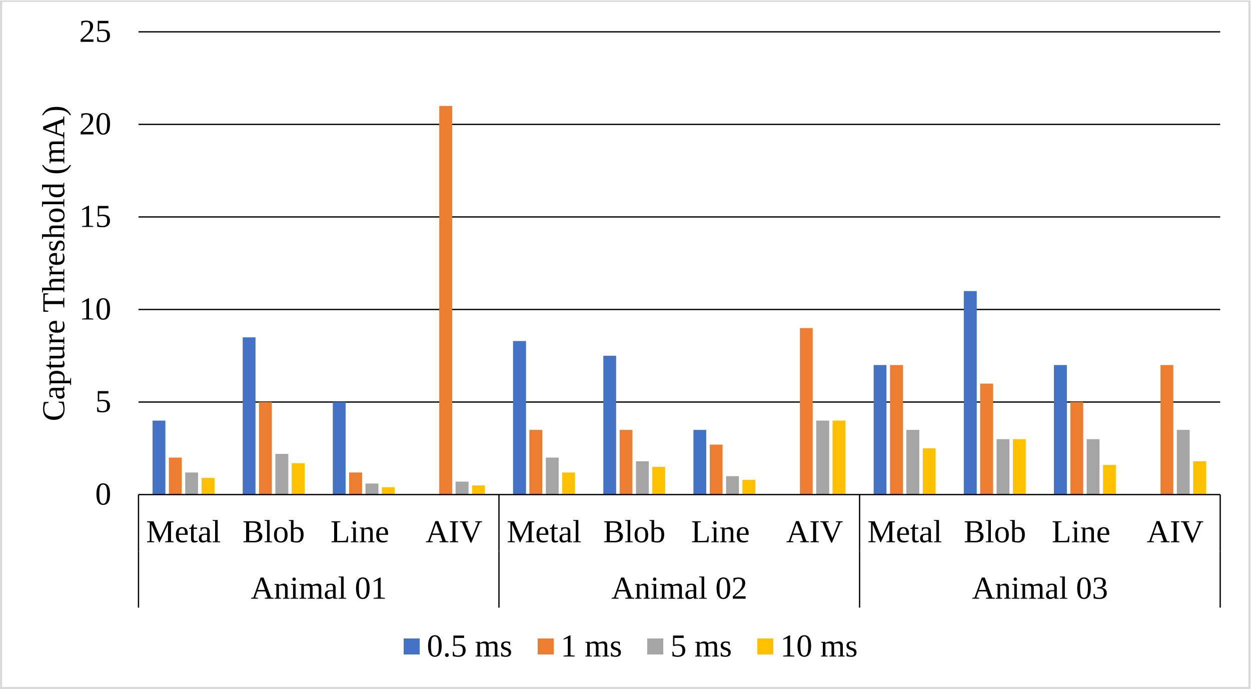

Supplementary Figure 14. Capture thresholds for each acute porcine study (n=3 pigs). Each bar represents a single value. Replicates are shown as Animal 01, 02 and 03. Source data are provided as a Source Data file.

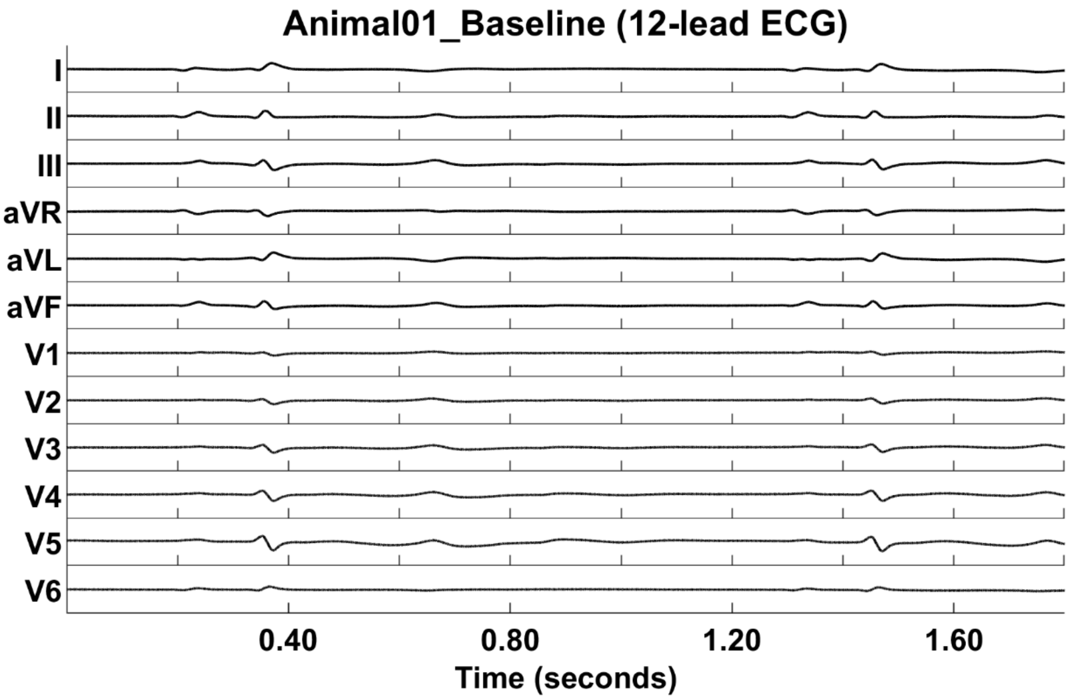

Supplementary Figure 15. 12-lead ECG of the heart at sinus rhythm. Source data are provided as a Source Data file.

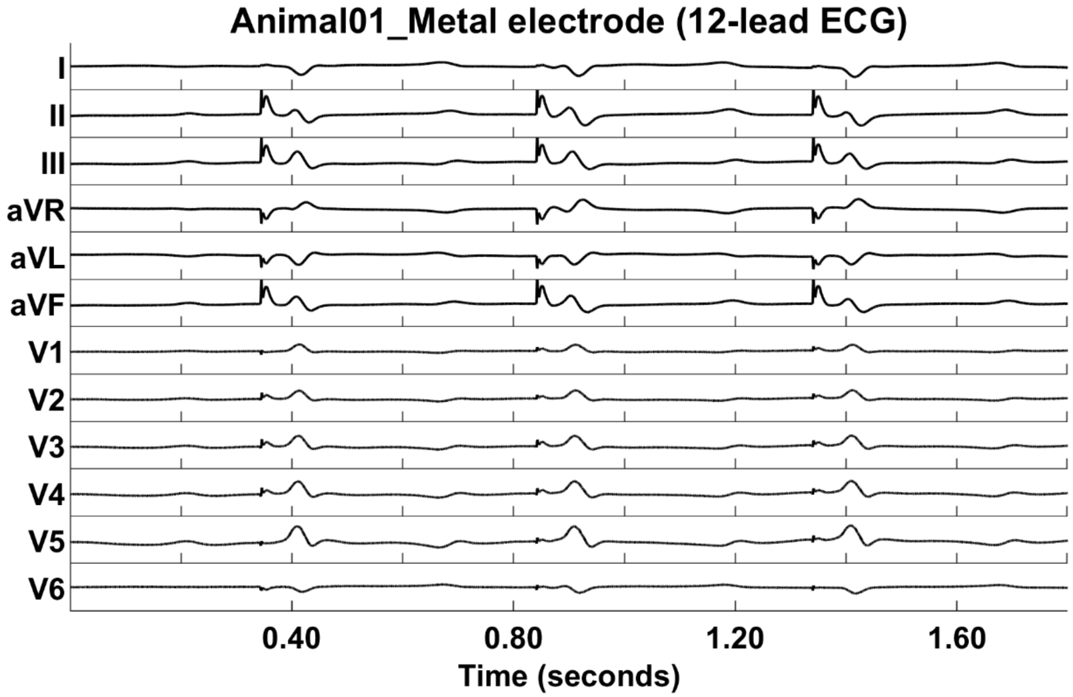

Supplementary Figure 16. 12-lead ECG of unipolar pacing with a metal electrode placed directly on the left ventricular myocardium. Pacing artifacts can be seen as spikes in each of the channels. Pacing was performed at 120 bpm. Source data are provided as a Source Data file.

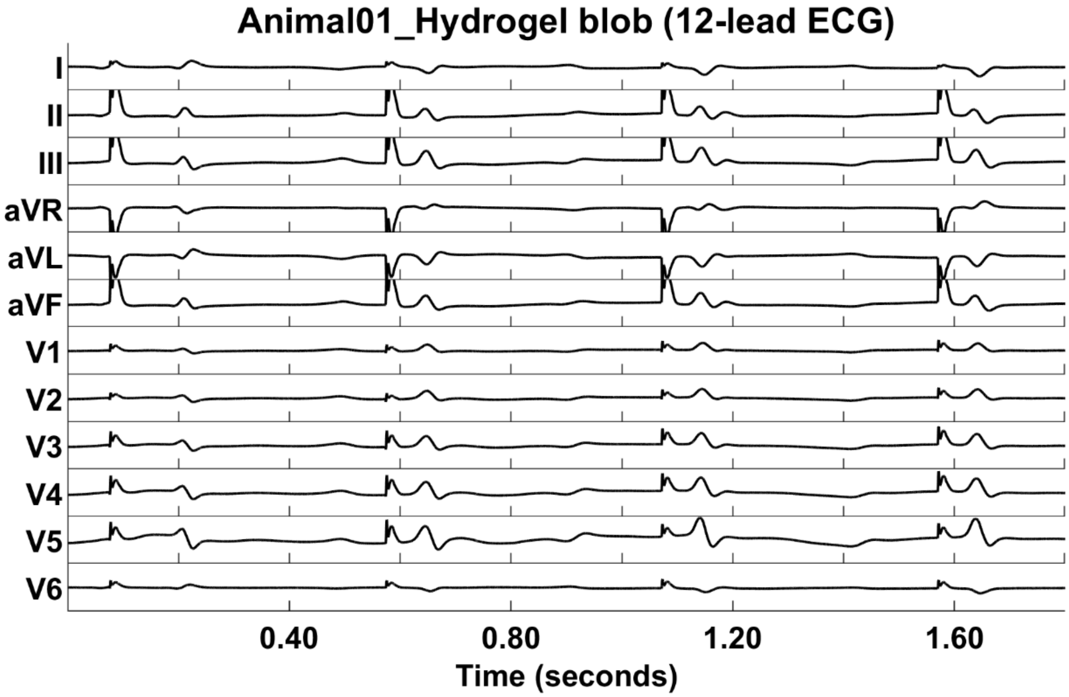

Supplementary Figure 17. 12-lead ECG of unipolar pacing with a small blob of hydrogel electrode placed directly on the left ventricular myocardium. Pacing artifacts can be seen as spikes in each of the channels. Pacing was performed at 120 bpm. Source data are provided as a Source Data file.

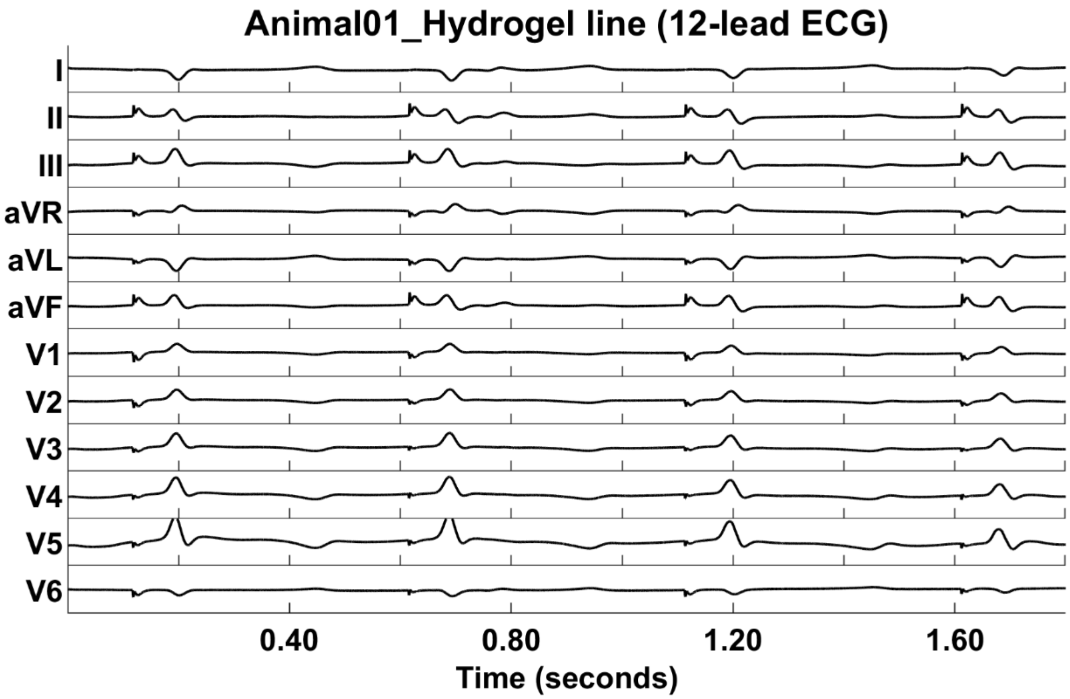

Supplementary Figure 18. 12-lead ECG of unipolar pacing with a linear hydrogel electrode (~4 cm) placed directly on the left ventricular myocardium close to the interventricular septum. Pacing artifacts can be seen as spikes in each of the channels. Pacing was performed at 120 bpm. Source data are provided as a Source Data file.

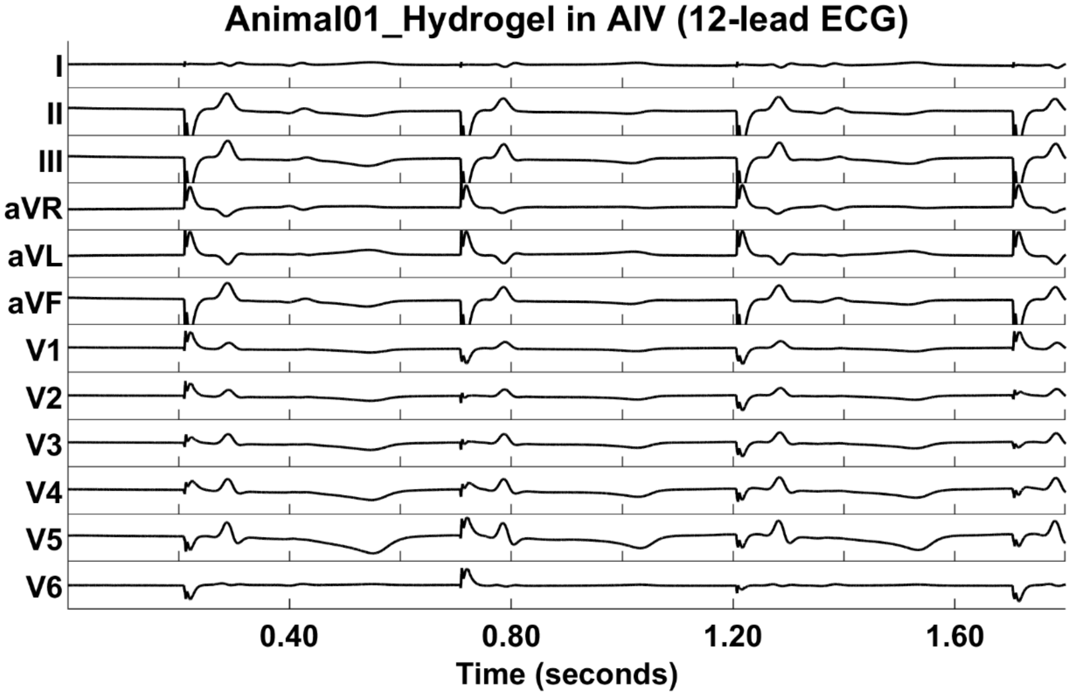

Supplementary Figure 19. 12-lead ECG of unipolar pacing from hydrogel cured in situ in the AIV. Pacing artifacts can be seen as spikes in each of the channels. Pacing was performed at 120 bpm. Source data are provided as a Source Data file.

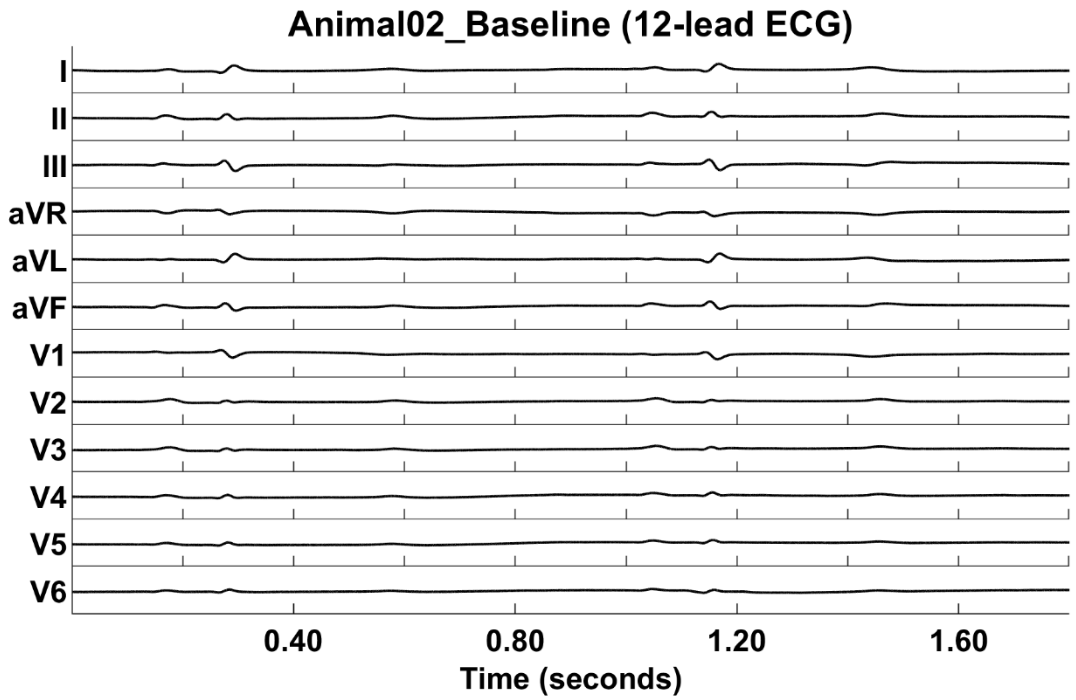

Supplementary Figure 20. Animal02: 12-lead ECG of the heart at sinus rhythm. Source data are provided as a Source Data file.

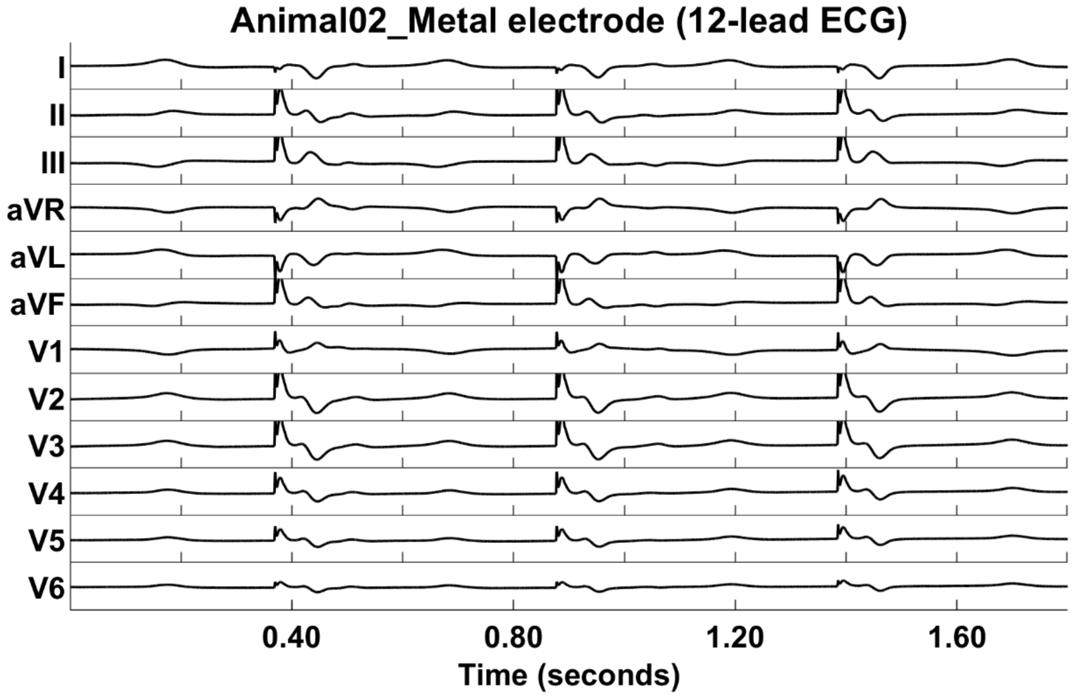

Supplementary Figure 21. Animal02: 12-lead ECG of unipolar pacing with a metal electrode placed directly on the left ventricular myocardium. Pacing artifacts can be seen as spikes in each of the channels. Pacing was performed at 120 bpm. Source data are provided as a Source Data file.

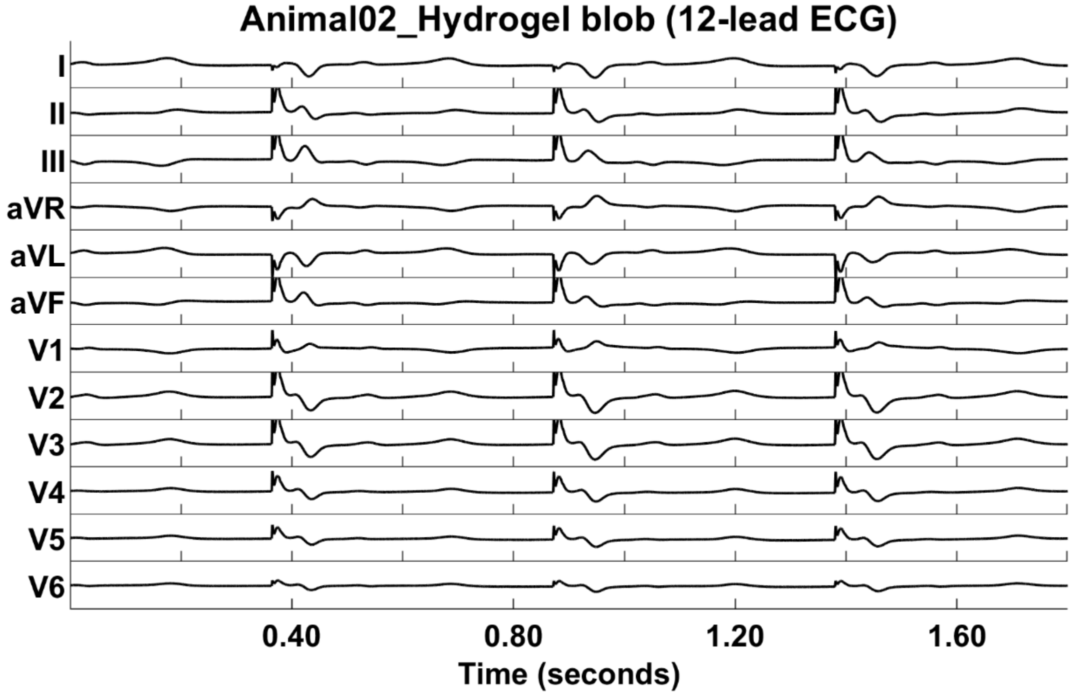

Supplementary Figure 22. Animal02: 12-lead ECG of unipolar pacing with a small blob of hydrogel electrode placed directly on the left ventricular myocardium. Pacing artifacts can be seen as spikes in each of the channels. Pacing was performed at 120 bpm. Source data are provided as a Source Data file.

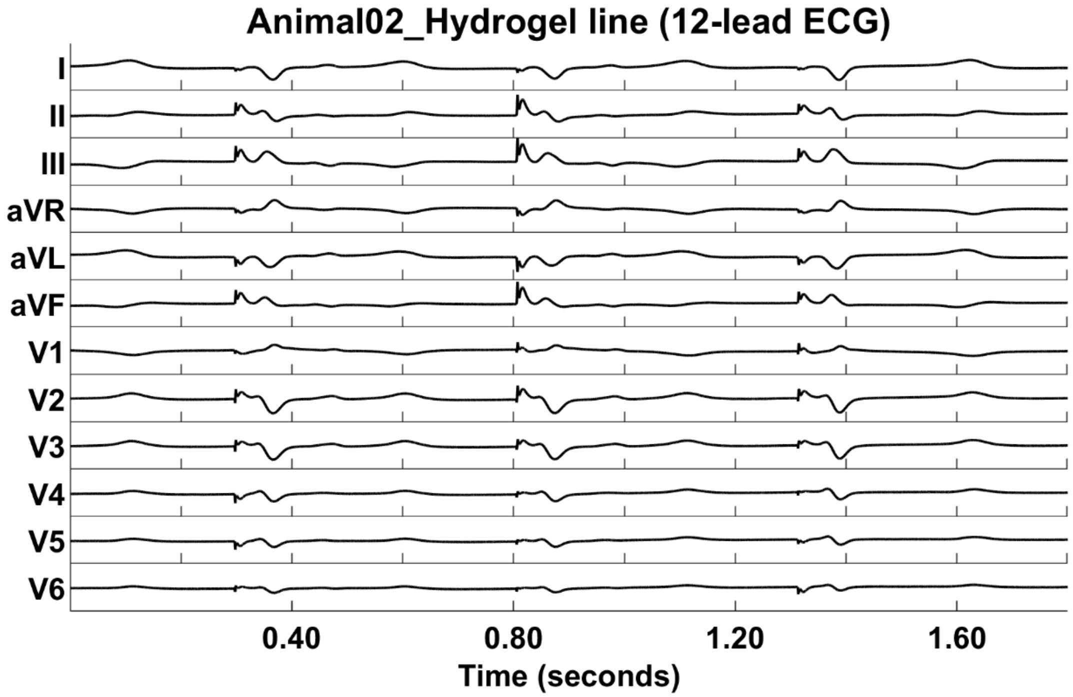

Supplementary Figure 23. Animal02: 12-lead ECG of unipolar pacing with a linear hydrogel electrode (~4 cm) placed directly on the left ventricular myocardium close to the interventricular septum. Pacing artifacts can be seen as spikes in each of the channels. Pacing was performed at 120 bpm. Source data are provided as a Source Data file.

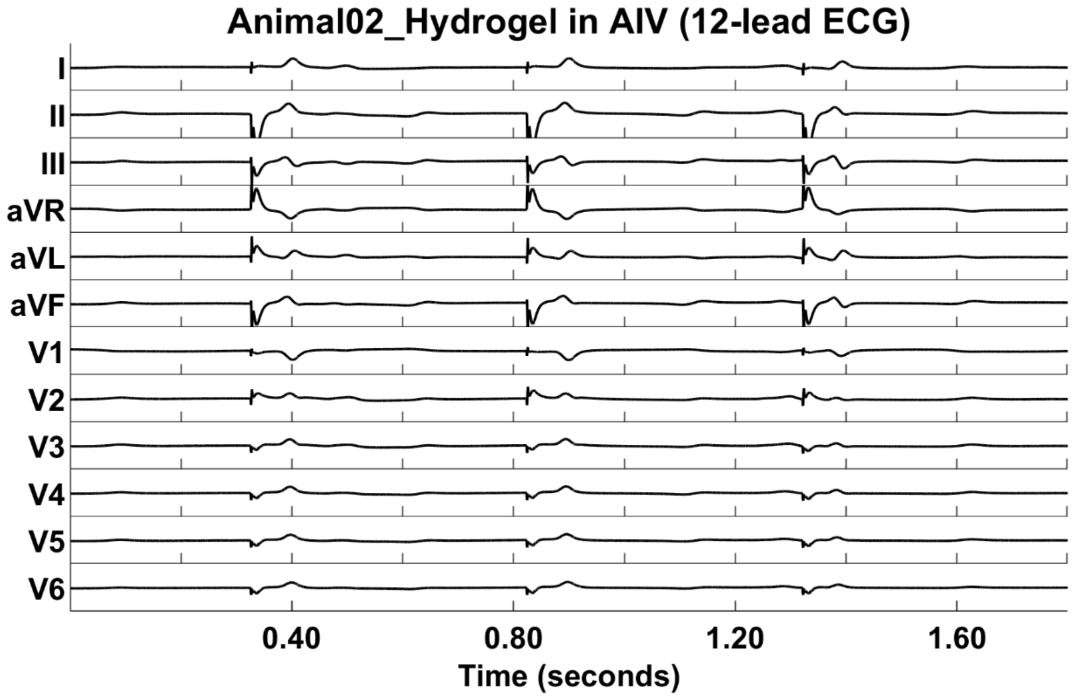

Supplementary Figure 24. Animal02: 12-lead ECG of unipolar pacing from hydrogel cured in situ in the AIV. Pacing artifacts can be seen as spikes in each of the channels. Pacing was performed at 120 bpm. Source data are provided as a Source Data file.

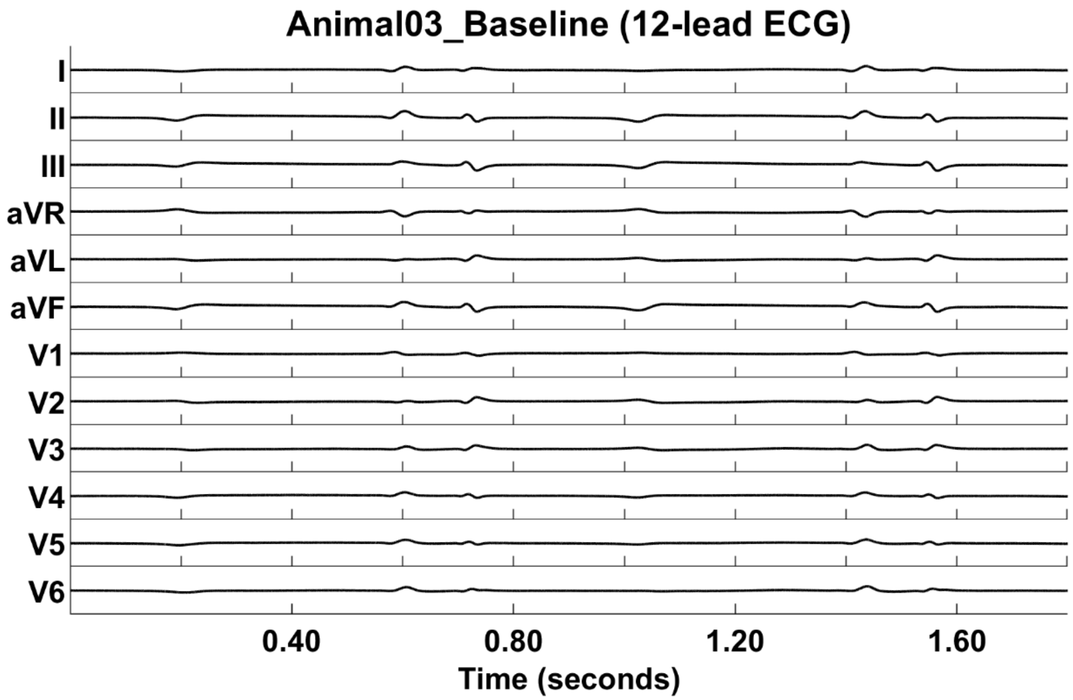

Supplementary Figure 25. Animal03: 12-lead ECG of the heart at sinus rhythm. Source data are provided as a Source Data file.

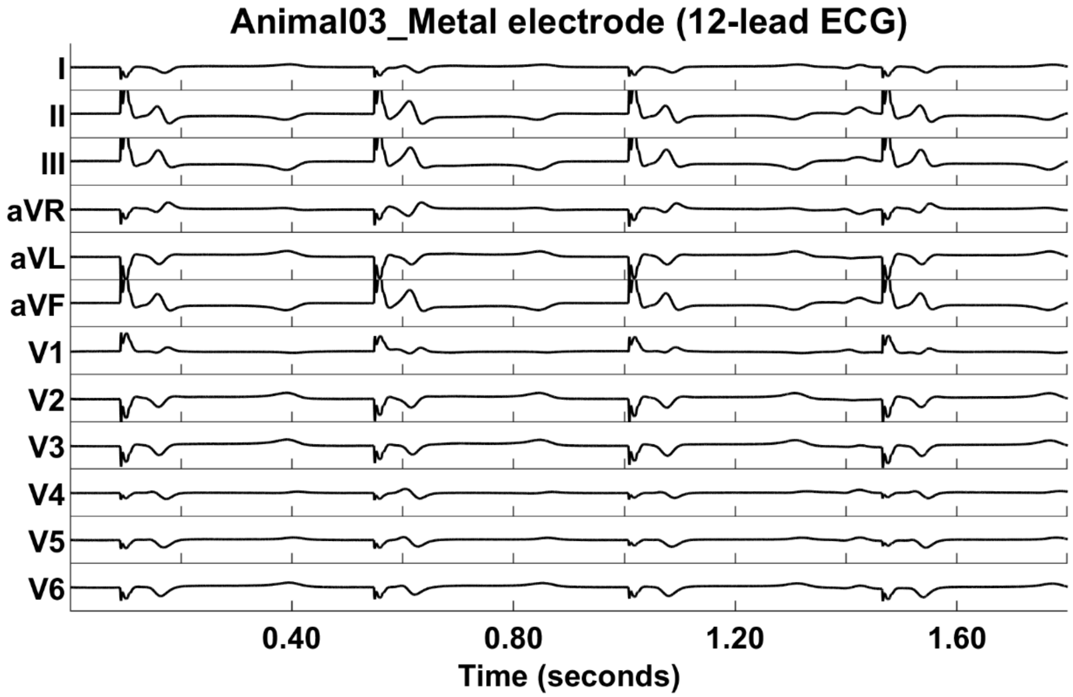

Supplementary Figure 26. Animal03: 12-lead ECG of unipolar pacing with metal electrode placed directly on the left ventricular myocardium. Pacing artifacts can be seen as spikes in each of the channels. Pacing was performed at 120 bpm. Source data are provided as a Source Data file.

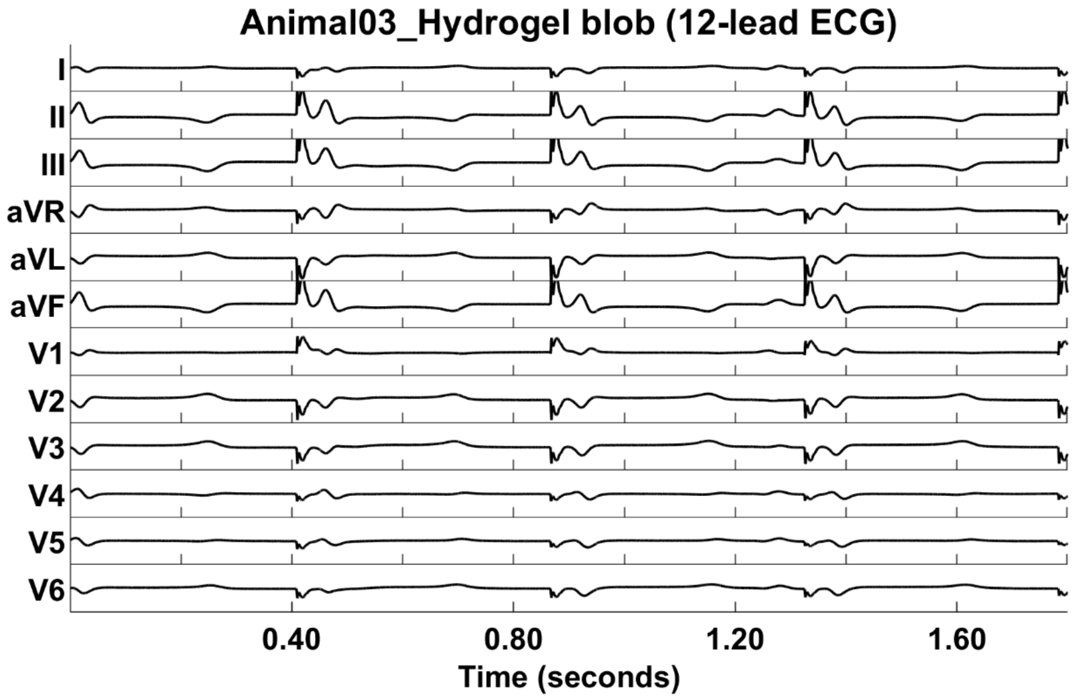

Supplementary Figure 27. Animal03: 12-lead ECG of unipolar pacing with a small blob of hydrogel electrode placed directly on the left ventricular myocardium. Pacing artifacts can be seen as spikes in each of the channels. Pacing was performed at 120 bpm. Source data are provided as a Source Data file.

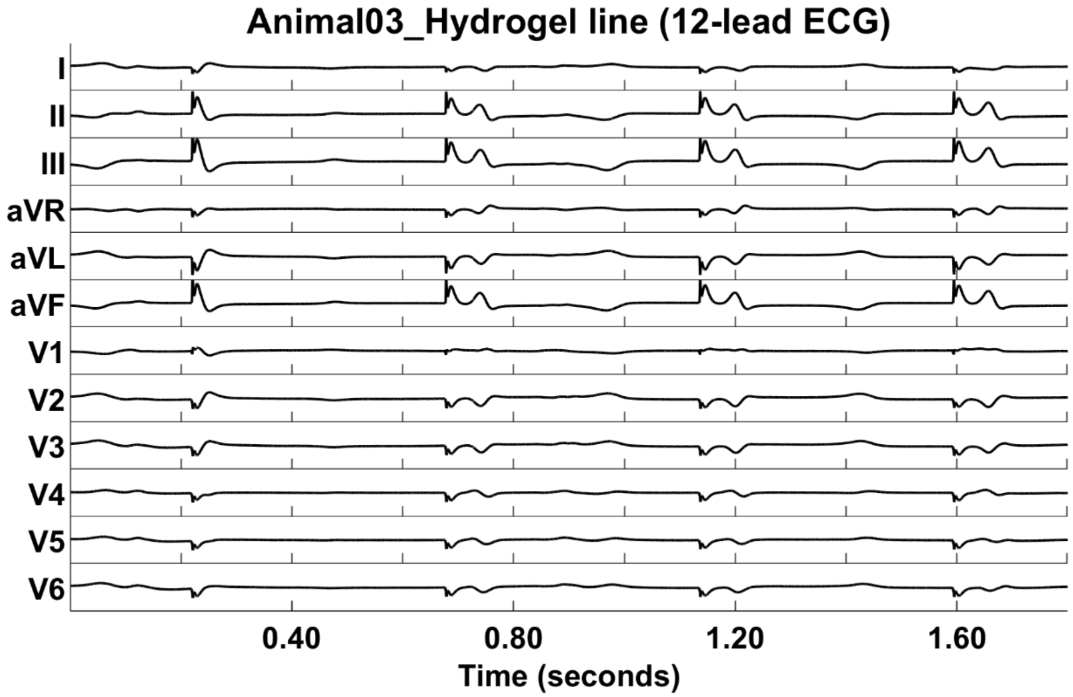

Supplementary Figure 28. Animal03: 12-lead ECG of unipolar pacing with a linear hydrogel electrode (~4 cm) placed directly on the left ventricular myocardium close to the interventricular septum. Pacing artifacts can be seen as spikes in each of the channels. Pacing was performed at 120 bpm. Source data are provided as a Source Data file.

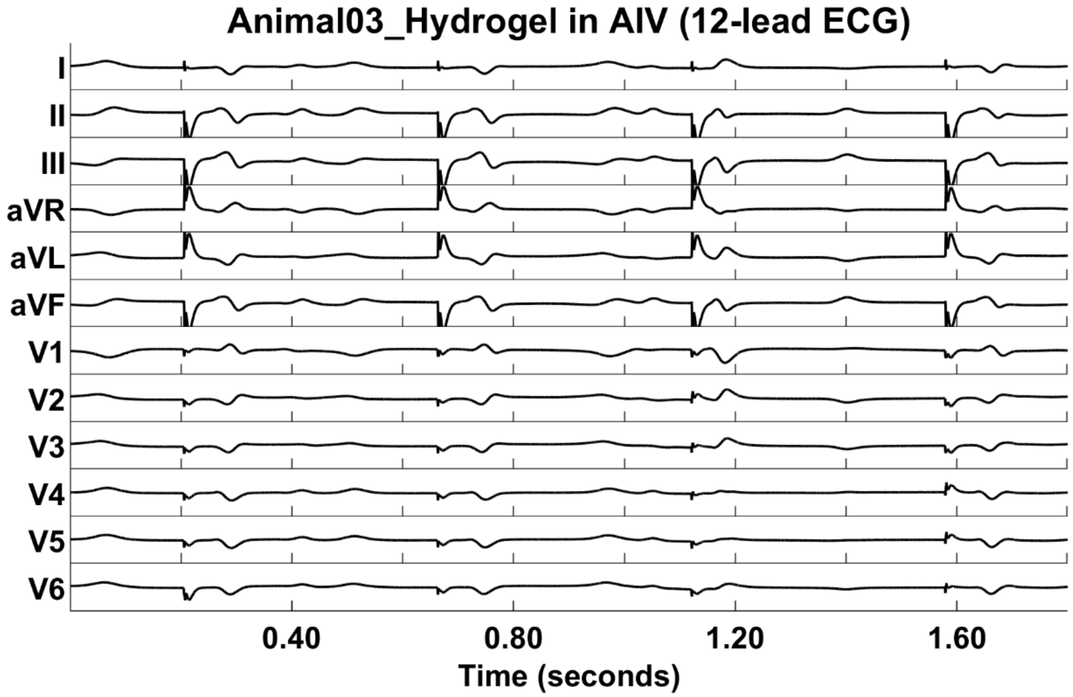

Supplementary Figure 29. Animal03: 12-lead ECG of unipolar pacing from hydrogel cured in situ in the AIV. Pacing artifacts can be seen as spikes in each of the channels. Pacing was performed at 120 bpm. Source data are provided as a Source Data file.

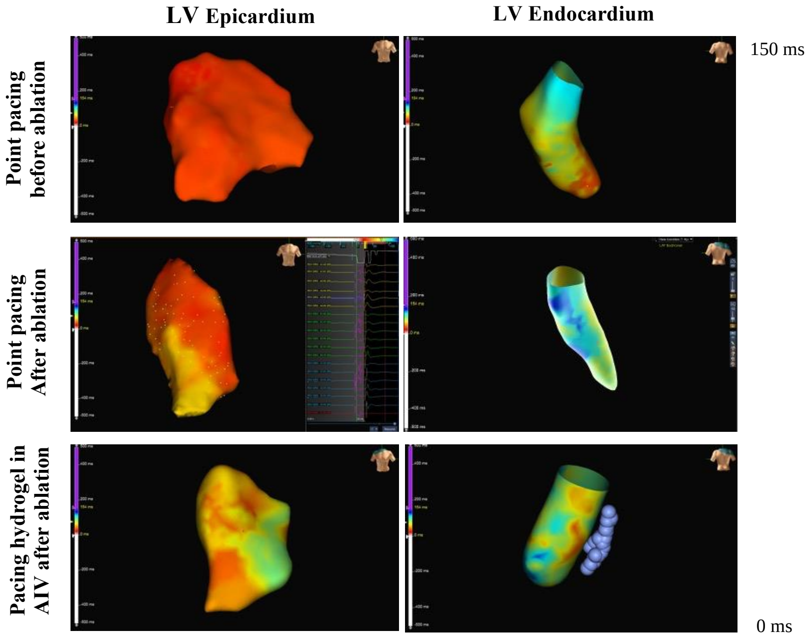

Supplementary Figure 30. Epicardial and endocardial electroanatomical mapping for animal AIV 1. The AIV hydrogel pacing is indicated by the blue circles.

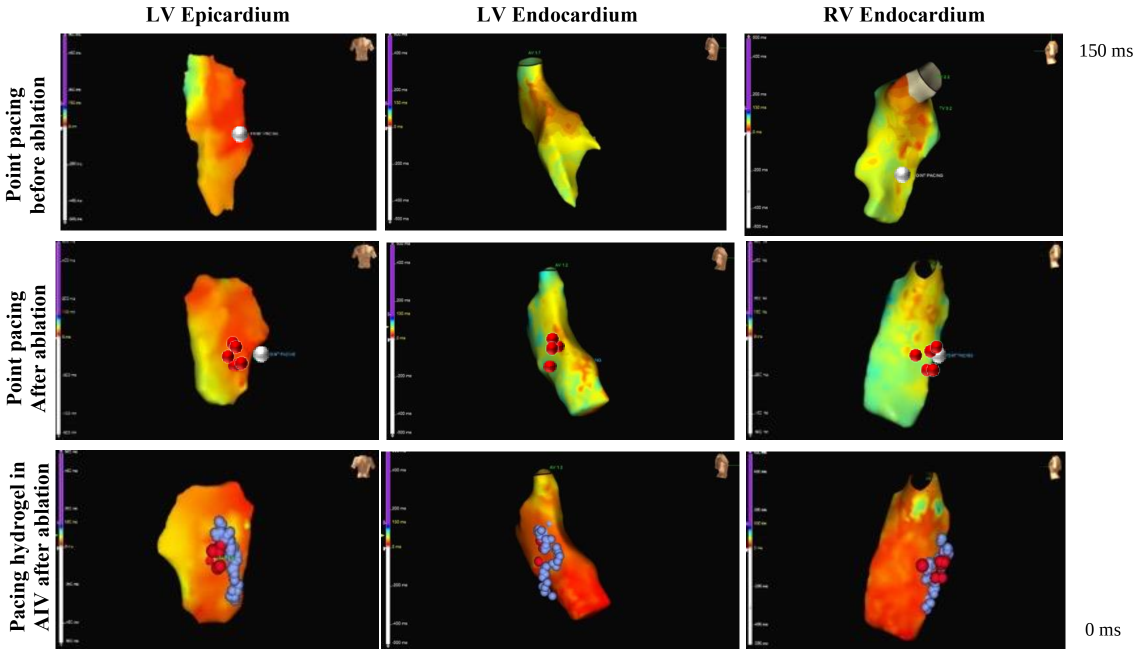

Supplementary Figure 31. Epicardial and endocardial electroanatomical mapping for animal AIV 2. Point pacing location is indicated by a small white circle, and the AIV hydrogel pacing is indicated by the blue circles. Points of ablation are marked by the red circles.

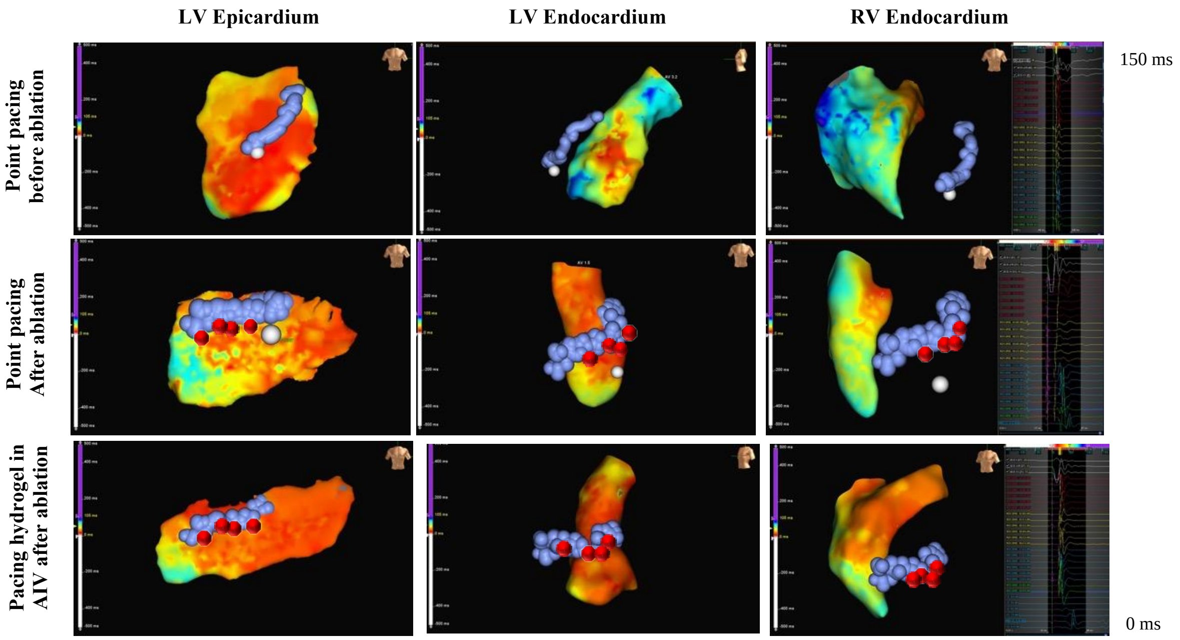

Supplementary Figure 32. Epicardial and endocardial electroanatomical mapping for animal AIV 3. Point pacing location is indicated by a small white circle, and the AIV is indicated by the blue circles. Points of ablation are marked by the red circles.

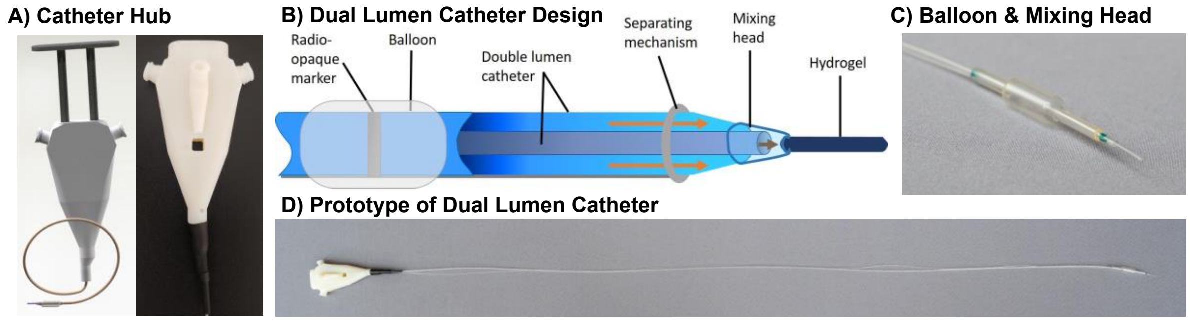

Supplementary Figure 33. Prototype of dual-lumen catheter. Schematic of the dual lumen catheter with catheter hub and mixing head for transcatheter delivery of in situ-curing hydrogel.

Supplementary Table 1. High sensitivity Troponin I measurements. Measurements from four separate studies from two weeks after occlusion of the MCV and from four weeks after occlusion of the AIV in a porcine model. The normal range for troponin I is from 3 to 70 ng.

Vein - Animal	Troponin levels (ng/L)					
	Pre-Op	Post-Op	Week 1	Week 2	Week 3	Week 4
**MCV - 1**	--	3313	--	34	--	--
**AIV - 1**	17	421	99	97	99	49
**AIV - 2**	121	N/A	202	95	116	54
**AIV - 3**	17	140	44	28	62	45

Supplementary Table 2. Capture thresholds (in mA) for each material and acute animal tested (n=3). NC = no capture.

PW (ms)	Animal 01				Animal 02				Animal 03			
	Metal	Blob	Line	AIV	Metal	Blob	Line	AIV	Metal	Blob	Line	AIV
0.5	4	8.5	5	-	8.3	7.5	3.5	-	7	11	7	-
1	2	5	1.2	21	3.5	3.5	2.7	9	7	6	5	7
5	1.2	2.2	0.6	0.7	2	1.8	1	4	3.5	3	3	3.5
10	0.9	1.7	0.4	0.5	1.2	1.5	0.8	4	2.5	3	1.6	1.8

Supplementary Table 3. Animal information for all specimens for acute and chronic in vivo studies.

Study	Duration	Name	Sex	Weight (lbs)	Age
Deployment & Pacing	Acute	Animal01	M	102	3m25d
Deployment & Pacing	Acute	Animal02	M	100	3m29d
Deployment & Pacing	Acute	Animal03	M	99	4m27d
Voltage Mapping	Acute	AIV 1	M	104	4m8d
Voltage Mapping	Acute	AIV 2	M	143	6m28d
Voltage Mapping	Acute	AIV 3	F	111	4m5d
Chronic Safety	Chronic	MCV	M	94	3m20d
Chronic Safety	Chronic	AIV 1	M	101	3m22d
Chronic Safety	Chronic	AIV 2	F	127	4m9d
Chronic Safety	Chronic	AIV 3	M	136	4m23d
