# Supplementary material for: Injectable hydrogel electrodes as conduction highways to restore native pacing
Source: Nat Commun. 2024 Jan 2;15:64. doi: 10.1038/s41467-023-44419-0 (PMC10762156; doi:10.1038/s41467-023-44419-0)
Supplement: Supplementary file 3 — Description of Additional Supplementary Files [file 41467_2023_44419_MOESM3_ESM.pdf]

Description of Additional Supplementary Files

Supplementary Movie 1. Echocardiogram 2 weeks after hydrogel injection into MCV in a pig model. Echocardiograms show no evidence of regional wall motion abnormalities after two weeks.

Supplementary Movie 2. Echocardiogram at baseline before hydrogel injection into the AIV in a pig model.

Supplementary Movie 3. Echocardiogram 4 weeks after hydrogel injection into the AIV in a pig model. Echocardiograms show no evidence of regional wall motion abnormalities at four weeks compared to baseline imaging.
