# Supplementary material for: Injectable hydrogel electrodes as conduction highways to restore native pacing
Source: Nat Commun. 2024 Jan 2;15:64. doi: 10.1038/s41467-023-44419-0 (PMC10762156; doi:10.1038/s41467-023-44419-0)
Supplement: Supplementary file 7 — Reporting Summary [file 41467_2023_44419_MOESM7_ESM.pdf]

nature portfolio

Corresponding author(s): Elizabeth Cosgriff-Hernandez, Mehdi Razavi

Last updated by author(s): Oct 23, 2023

Reporting Summary

Nature Portfolio wishes to improve the reproducibility of the work that we publish. This form provides structure for consistency and transparency in reporting. For further information on Nature Portfolio policies, see our Editorial Policies and the Editorial Policy Checklist.

Statistics

For all statistical analyses, confirm that the following items are present in the figure legend, table legend, main text, or Methods section.

n/a	Confirmed	
☐	☒	The exact sample size (*n*) for each experimental group/condition, given as a discrete number and unit of measurement
☐	☒	A statement on whether measurements were taken from distinct samples or whether the same sample was measured repeatedly
☐	☒	The statistical test(s) used AND whether they are one- or two-sided<br>*Only common tests should be described solely by name; describe more complex techniques in the Methods section.*
☐	☒	A description of all covariates tested
☐	☒	A description of any assumptions or corrections, such as tests of normality and adjustment for multiple comparisons
☐	☒	A full description of the statistical parameters including central tendency (e.g. means) or other basic estimates (e.g. regression coefficient) AND variation (e.g. standard deviation) or associated estimates of uncertainty (e.g. confidence intervals)
☐	☒	For null hypothesis testing, the test statistic (e.g. *F*, *t*, *r*) with confidence intervals, effect sizes, degrees of freedom and *P* value noted<br>*Give P values as exact values whenever suitable.*
☒	☐	For Bayesian analysis, information on the choice of priors and Markov chain Monte Carlo settings
☒	☐	For hierarchical and complex designs, identification of the appropriate level for tests and full reporting of outcomes
☒	☐	Estimates of effect sizes (e.g. Cohen's *d*, Pearson's *r*), indicating how they were calculated

Our web collection on statistics for biologists contains articles on many of the points above.

Software and code

Policy information about availability of computer code

Data collection	N/A. No custom software used for data collection
Data analysis	N/A. No custom code was used to process data.

For manuscripts utilizing custom algorithms or software that are central to the research but not yet described in published literature, software must be made available to editors and reviewers. We strongly encourage code deposition in a community repository (e.g. GitHub). See the Nature Portfolio guidelines for submitting code & software for further information.

Data

Policy information about availability of data

All manuscripts must include a data availability statement. This statement should provide the following information, where applicable:

- Accession codes, unique identifiers, or web links for publicly available datasets
- A description of any restrictions on data availability
- For clinical datasets or third party data, please ensure that the statement adheres to our policy

All data are available in the main text or the supplementary materials. A file with all the source data was submitted with this manuscript. All the raw data will be uploaded into The University of Texas at Austin UTBox cloud storage. Access to the data will be provided upon request.

Research involving human participants, their data, or biological material

Policy information about studies with human participants or human data. See also policy information about sex, gender (identity/presentation), and sexual orientation and race, ethnicity and racism.

Reporting on sex and gender	N/A
Reporting on race, ethnicity, or other socially relevant groupings	N/A
Population characteristics	N/A
Recruitment	N/A
Ethics oversight	N/A

Note that full information on the approval of the study protocol must also be provided in the manuscript.

Field-specific reporting

Please select the one below that is the best fit for your research. If you are not sure, read the appropriate sections before making your selection.

☒ Life sciences ☐ Behavioural & social sciences ☐ Ecological, evolutionary & environmental sciences

For a reference copy of the document with all sections, see nature.com/documents/nr-reporting-summary-flat.pdf

Life sciences study design

All studies must disclose on these points even when the disclosure is negative.

Sample size	Minimum sample size for conductivity retention was estimated based on power analysis using in vitro data. Sample size for safety assessment and electrical mapping was decided to be only n=3, to demonstrate reproducibility as a proof of concept.
Data exclusions	No data was excluded for the analysis
Replication	Safety assessment, in vivo hydrogel formation and electrical mapping studies were done in three different pigs (n=3, biological replicates). The data shown successful replicates is included in the manuscript and SI.
Randomization	No need for randomization, since we were not exploring multiple treatments. No need for covariates since all studies were replicates perform on different days by the same technicians. For pacing experiments, all modalities were tested on each animal.
Blinding	Blinding was not relevant for these studies since we were showing reproducibility and all were treated in the same way.

Reporting for specific materials, systems and methods

We require information from authors about some types of materials, experimental systems and methods used in many studies. Here, indicate whether each material, system or method listed is relevant to your study. If you are not sure if a list item applies to your research, read the appropriate section before selecting a response.

Materials & experimental systems

n/a	Involved in the study	
☒	☐	Antibodies
☒	☐	Eukaryotic cell lines
☒	☐	Palaeontology and archaeology
☐	☒	Animals and other organisms
☒	☐	Clinical data
☒	☐	Dual use research of concern
☒	☐	Plants

Methods

n/a	Involved in the study	
☒	☐	ChIP-seq
☒	☐	Flow cytometry
☒	☐	MRI-based neuroimaging

Animals and other research organisms

Policy information about studies involving animals; ARRIVE guidelines recommended for reporting animal research, and Sex and Gender in Research

Laboratory animals	Detailed information about animals provided on Supplementary Table 3. Safety assessment and cardiac electroanatomical mappings studies were done using Yorkshire pigs between 40kg and 50kgs. Pigs age was between 3 months+20 days and 6 months+28days.
Wild animals	The study did not involve wild animals
Reporting on sex	Sex differences not considered for this study. Proof of concept was done with n=3. Future studies with larger samples size will consider impact of sex.
Field-collected samples	The study did not involve samples collected from the field.
Ethics oversight	All procedures were approved by the Institutional Animal Care & Use Committee (IACUC) at the Texas Heart Institute.

Note that full information on the approval of the study protocol must also be provided in the manuscript.
